# Eliminating the capsule-like layer to promote glucose uptake for hyaluronan production by engineered *Corynebacterium glutamicum*

**DOI:** 10.1038/s41467-020-16962-7

**Published:** 2020-06-19

**Authors:** Yang Wang, Litao Hu, Hao Huang, Hao Wang, Tianmeng Zhang, Jian Chen, Guocheng Du, Zhen Kang

**Affiliations:** 1 The Key Laboratory of Carbohydrate Chemistry and Biotechnology, Ministry of Education, Jiangnan University, 214122 Wuxi, China; 2 The Key Laboratory of Industrial Biotechnology, Ministry of Education, School of Biotechnology, Jiangnan University, 214122 Wuxi, China; 3 Bloomage Biotechnology CO, LTD, 250000 Jinan, China

**Keywords:** Polysaccharides, Industrial microbiology, Metabolic engineering, Applied microbiology

## Abstract

Hyaluronan is widely used in cosmetics and pharmaceutics. Development of robust and safe cell factories and cultivation approaches to efficiently produce hyaluronan is of many interests. Here, we describe the metabolic engineering of *Corynebacterium glutamicum* and application of a fermentation strategy to manufacture hyaluronan with different molecular weights. *C. glutamicum* is engineered by combinatorial overexpression of type I hyaluronan synthase, enzymes of intermediate metabolic pathways and attenuation of extracellular polysaccharide biosynthesis. The engineered strain produces 34.2 g L^−1^ hyaluronan in fed-batch cultures. We find secreted hyaluronan encapsulates *C. glutamicum*, changes its cell morphology and inhibits metabolism. Disruption of the encapsulation with leech hyaluronidase restores metabolism and leads to hyper hyaluronan productions of 74.1 g L^−1^. Meanwhile, the molecular weight of hyaluronan is also highly tunable. These results demonstrate combinatorial optimization of cell factories and the extracellular environment is efficacious and likely applicable for the production of other biopolymers.

## Abstract (web summary)

Bioproduction of hyaluronan needs increases in yield and greater diversity of the molecular weights. Here, the author increases hyaluronan production and diversifies the molecular weights through engineering the hyaluronan biosynthesis pathway and disruption of *Corynebacterium glutamicum* encapsulation caused by secreted hyaluronan.

## Introduction

Hyaluronan (hyaluronic acid or HA) is a negatively charged, non-sulfated glycosaminoglycan comprising repeating uridine diphosphate glucuronate (UDP-GlcA) and uridine diphosphate N-acetylglucosamine (UDP-GlcNAc) disaccharide units. It is a natural substance in vertebrates and mainly found in the eyes, joints and skin, where it absorbs large amounts of water for joint lubrication^1^, cell coating or repair of damaged skin tissue^2^. In addition to vertebrates, some pathogenic microorganisms, such as group A *Streptococcus*^3^ and *Pasteurella multocida* type A strains^4^, produce HA as the major component of their capsules for protection against exterior damage. Due to its biocompatibility, hygroscopicity and non-immunogenicity, HA and its derivatives are important to the cosmetics, pharmaceutical, biomedical, and food industries^5–8^.

Currently, commercial production of HA is mainly dependent on fermentation of group A *Streptococcus*^9^; however, high risk of pathogenicity to livestock and contamination by exotoxins in the HA product hinder its broader application. The development of metabolic engineering allows the engineering of non-pathogenic *Escherichia coli* strains for heterologous HA production^10,11^. To further eliminate potential safety concerns, many generally recognized as safe (GRAS) strains, such as *Bacillus subtilis*^12–16^, *Lactococcus lactis*^17–20^, *Corynebacterium glutamicum*^21,22^, and *Pichia pastoris*^23^, have been engineered as alternative producers of HA. Additionally, cell-free systems have been exploited to produce HA at specific molecular weight (MW)^24,25^. Various strategies have been adopted to construct these HA producers, with selection of HA synthases and host strains representing the most critical step, as variations in HA synthase sequence, structural conformation^26^, or host cell metabolic capacities make differences in HA yield or MW. HA synthase activities have been improved by protein engineering^24,27^ or modification of the microenvironment of the enzymatic reaction, including the membrane lipid composition^28^. Moreover, metabolic engineering strategies such as overexpressing enzymes of intermediate metabolic pathways (e.g., UDP-glucose 6-dehydrogenase or glucosamine-1-phosphate N-acetyltransferase) or blocking the synthesis of unwanted metabolites (e.g., l-lactate) were adopted to drive the generation of intermediate metabolites required for HA synthesis^29,30^.

In view of the GRAS status and metabolic capacity, *C. glutamicum* as the Gram-positive model organism has been engineered as an HA producer. In 2014, Hoffmann et al.^21^ first constructed the HA biosynthetic pathway by expressing *Streptococcus equi* subsp. *zooepidemicus* HA synthase (seHasA) in *C. glutamicum*. The engineered strain produced 1.2 g L^−1^ of HA. In 2016, Cheng et al.^31^ co-expressed the codon-optimized *Streptococcus dysgalactiae* subsp. *equisimilis ssehasA* gene along with endogenous *ugdA* in *C. glutamicum* to produce 8.3 g L^−1^ HA in fed-batch cultivation. Additionally, deletion of *ldh*, encoding lactate dehydrogenase, increased HA production to 21.6 g L^−1^ in fed-batch culture^29^. A follow-up study enhanced the HA yield to 28.7 g L^−1^ by attenuating the glycolysis pathway, pentose phosphate pathway and the dehydrogenation of pyruvate^22^.

In the present study, we engineer *C. glutamicum* for high-yield HA production by selecting the most productive HA synthase, overexpressing enzymes of the intermediate pathways to convert glucose into the HA building blocks UDP-GlcA and UDP-GlcNAc and decreasing endogenous extracellular polysaccharide biosynthesis. The engineered strain produces 34.2 g L^−1^ HA in fed-batch cultures. Our analysis of cell morphology reveals that the secreted HA forms an HA capsule-like layer, which is subsequently found to restrict nutrient uptake and inhibit HA synthesis. To relieve this inhibition effect, we supplement leech hyaluronidase (LHYal, hydrolase)^32^ to the fed-batch culture to disrupt cell encapsulation and decrease broth viscosity. This strategy significantly promotes glucose uptake and HA production.

## Results

### Construction of HA-producing *C. glutamicum* strains

**Fig. 1:**
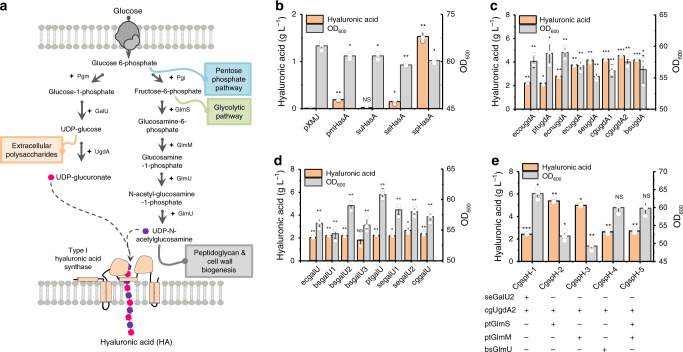
Engineering *C. glutamicum* for HA biosynthesis from glucose. **a** HA chains are elongated by HA synthase (HasA) from two building blocks: UDP-GlcA (uridine diphosphate glucuronate) and UDP-GlcNAc (uridine diphosphate N-acetylglucosamine). Pgm, phosphoglucomutase; GalU, glucose-1-phosphate uridylyltransferase; UgdA, UDP‐glucose 6‐dehydrogenase; Pgi, glucose-6-phosphate isomerase; GlmS, l-glutamine-d-fructose-6-phosphate aminotransferase; GlmM, phosphoglucosamine mutase; GlmU, UDP-N-acetylglucosamine pyrophosphorylase/Glucosamine-1-phosphate N-acetyltransferase. **b** HA synthesis by *P. multocida* (pmHasA), *S. pyogenes* (spHasA), *S. uberis* (suHasA), and *S. equi* subsp. *zooepidemicus* HasA (seHasA), respectively in *C. glutamicum*. **c**, **d** Comparison of the activities of UgdA and GalU from different species on enhancing HA production. **e** Co-overexpression of *C. glutamicum* cgUgdA2 with *S. equi* subsp. *zooepidemicus* seGalU2, *P. putida* ptGlmS, *P. putida* ptGlmM or *B. subtilis* bsGlmU to enhance HA production. All the data are expressed as the mean ± S.D. from three (*n* = 3) biologically independent replicates. Statistical evaluation (*p*-value) compared to strain pXMJ (**a**), spHasA (**b**, **c**), or cgugdA2 (**e**) was performed by two-sided *t*-test. *p < 0.05, **p < 0.01, ***p < 0.001; NS not significant (*p* ≥ 0.05). Source data underlying Fig. 1b–e are provided as a Source data file.

HA synthase polymerizes HA chains using two building blocks: UDP-GlcA and UDP-GlcNAc (Fig. 1a). Two types of bacterial HA synthases have been identified: type I synthase is a bifunctional enzyme that polymerizes the HA chain, as well as transfers HA across the cell membrane^33–35^ (Supplementary Fig. 1); and type II HA synthase, found only in *P. multocida*^36^, is a cytosolic glycosyltransferase with high affinity to the cytoplasmic membrane (Supplementary Fig. 1). Here, HA synthases from *P. multocida* (pmHasA), *S. pyogenes* (spHasA), *S. uberis* (suHasA), and* S. equi* subsp. *zooepidemicus* HasA (seHasA) were selected and overexpressed individually in the host strain *C. glutamicum* ATCC 13032. Comparatively, spHasA generated the highest HA titer (1.5 g L^−1^) in shake-flask cultivation, which was 10-fold and 7-fold higher than that of seHasA and pmHasA, respectively (Fig. 1b). Almost no HA was synthesized by suHasA (Fig. 1b). Protein alignment showed seHasA^21^ and sseHasA^22,29,31^ share the same protein sequence. In comparison, there is 72% sequence identity between seHasA and spHasA and 71% sequence identity between suHasA and spHasA (Supplementary Fig. 2a). Western Blot demonstrated suHasA expressed very poorly in *C. glutamicum* while the expression levels of spHasA and seHasA are similar (Supplementary Fig. 2b). To further explore the reason of the distinct HA synthesis capabilities between seHasA and spHasA, we replaced the most dissimilar region of seHasA from spHasA, the first transmembrane helix Leu7-Val25 of seHasA with the corresponding transmembrane helix Thr7-Met25 of spHasA^35^ to generate seHasA^Thr7-Met25^. The mutant seHasA^Thr7-Met25^ was expressed to the same level of the wild-type seHasA in *C. glutamicum* (Supplementary Fig. 2b). However, seHasA^Thr7-Met25^ produced much higher amount of HA (0.87 g L^−1^, Supplementary Fig. 2c), suggesting the first tranmembrane helix of type I HA synthase should play critical roles in regulating HA synthesis.

After determining spHasA as the best performing HA synthase, all genes encoding enzymes related to intermediate metabolite-synthesis pathways, including *galU*, *ugdA*, *glmS*, *glmM*, and *glmU*, from five species (*C. glutamicum* ATCC13032, cg; *B. subtilis* 168, bs; *S. equi* subsp. *zooepidemicus *WSH-24, se; *Pseudomonas putida* KT2440, pt; and *E. coli* MG1655, ec) were selected and individually overexpressed to examine their contributions to HA formation. In particular, *ugdA* genes (also known as *kfiD*) found in the heparosan-biosynthesis genome island of *E. coli* O10:K5:H4 ATCC 23506 (eco) and *E. coli* Nissle 1917 (ecn) (Supplementary Fig. 3) were investigated.

We found that UDP-glucose 6-dehydrogenase (UgdA) from different species exhibited different abilities to promote HA biosynthesis (4.5 g L^−1^, Fig. 1c). Endogenous UgdA encoded by *cgugdA2* in *C. glutamicum* showed the best performance in enhancing HA biosynthesis as compared with GalU from *S. equi* subsp. *zooepidemicus*, GlmS, GlmM from *P. putida* and GlmU from *B. subtilis*, which showed similar capabilities at stimulating HA biosynthesis (around 2 g L^−1^) (Fig. 1d and Supplementary Fig. 4a–c). Therefore, we overexpressed *cgugdA2* along with* S. equi* subsp. *zooepidemicus* *galU2* (*segalU2*), *P. putida glmS* (*ptglmS*) and *glmM* (*ptglmM*) and *B. subtilis glmU* (*bsglmU*), respectively, finding that co-overexpression of *cgugdA2* with *ptglmS* or *ptglmM* improved HA production to 5.4 g L^−1^ and 5.0 g L^−1^, respectively, in shake-flask cultivations (Fig. 1e). By contrast, other combinations failed to further improve the HA synthesis (Fig. 1e).

### Enhancement of HA production by attenuating the biosynthesis of extracellular polysaccharides

**Fig. 2:**
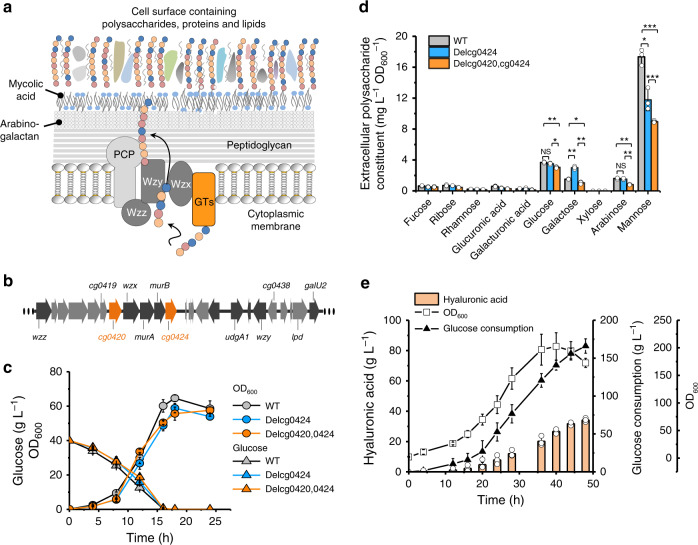
Deletion of *cg0420* and *cg0424* to improve HA production. **a** The model of the *C. glutamicum* cell envelope structure^38,39^. Extracellular polysaccharides/cell-surface polysaccharides, proteins and lipids are attached to the mycolic acid and arabinogalactan layer to form the outmost layer of the *C. glutamicum* cell wall. The Wzx/Wzy-dependent pathway predicted to participate in synthesis of cell-surface polysaccharides^41^. GTs, glycosyltransferases; PCP, polysaccharide co-polymerase. **b** Gene cluster putatively involved in synthesis of cell-surface polysaccharides. Genes *cg0419*, *cg0420*, *cg0424*, and *cg0438* found in close proximity to *wzx* and *wzy* were predicted to code glycosyltransferases. **c** Growth and metabolism of *C. glutamicum* ATCC 13032 (WT), *cg0424* deletion strain (Delcg0424) or *cg0420* and *cg0424* double deletion strain (Delcg0420,0424). **d** Sugar constituent of cell-surface polysaccharides isolated from *C. glutamicum* ATCC 13032 (WT), *cg0424* deletion strain (Delcg0424) and *cg0420* and *cg0424* double deletion strain (Delcg0420,0424). **e** Fed-batch cultivation of CgspH-7 in a 5-L fermenter. Real-time total consumed glucose, cell density (OD_600_), and HA yield were plotted. Seed culture was inoculated by 10% (v/v). Initial glucose content in the fed-batch culture was 40 g L^−1^. Post-inoculation glucose concentration was maintained at between 10 and 15 g L^−1^. Ammonia [14% (v/v)] was fed automatically to maintain pH at between 6.5 and 7.0 and air flow was maintained at 5 *vvm*. All the data are expressed as the mean ± S.D. from three (*n* = 3) biologically independent replicates. Each biological replicate of HA concentration assay was presented as the mean value of three technical repeats. Statistical evaluation (*p*-value) was performed by two-sided *t*-test. *p < 0.05, **p < 0.01, ***p < 0.001; NS not significant (*p* ≥ 0.05). Source data underlying Fig. 2c–e are provided as a Source data file.

We then investigated a pathway competing with HA synthesis, specifically the biosynthesis of extracellular polysaccharides, which is involved in consumption of key intermediate metabolites such as UDP-glucose^37^, the precursor of UDP-GlcA (Fig. 1a). The corynebacterial cell wall contains a peptidoglycan-attached arabinogalactan structure, which is enveloped by mycolic acid and an outer layer of extracellular polysaccharides, proteins and lipids^38^ (Fig. 2a). The main components of corynebacterial extracellular polysaccharides (also considered as capsular polysaccharides or cell-surface polysaccharides) are arabinomannan, mannan and glucan^38,39^. However, the genes required for biosynthesis of *C. glutamicum* cell-surface polysaccharides remain largely unknown. Taniguchi et al.^40^ reported that overexpression of *C. glutamicum* sigma factor SigD induced secretion of carbohydrate compounds and raised the expression levels of three glycosyltransferases genes (*cg0420*, *cg0532*, and *cg1181*). Following genome analysis, we found that *cg0420* locates in close proximity to the *wzx*, *wzy*, and *wzz* genes (Fig. 2b). Because the Wzx/Wzy-dependent assembly pathway synthesizes the majority of bacterial cell-surface polysaccharides (Fig. 2a)^41^, we speculated that *cg0420* may participate in biosynthesis of *C. glutamicum* extracellular polysaccharides. Prior to *cg0420* deletion, we found three other putative glycosyltransferase genes within the same genomic region (*cg0424*, *cg0419*, and *cg0438*) (Fig. 2b and Supplementary Fig. 5).

After many rounds of trials, we did not obtain single deletions for *cg0420*, *cg0419*, or *cg0438*. However, we were able to delete *cg0424* to generate strain Delcg0424. Basing on Delcg0424, we further deleted *cg0420* and constructed strain Delcg0420,0424. It was found that neither single *cg0424* deletion nor double deletion of *cg0420* and *cg0424* impaired cell growth (Fig. 2c). Sugar constituent analysis of the Delcg0424 extracellular polysaccharides showed that the concentration of mannose decreased by 32% while the concentration of arabinose was not affected (Fig. 2d). The concentrations of mannose and arabinose released from strain Delcg0420,0424 extracellular polysaccharides hydrolysis decreased by 24% and 47% respectively, comparing to that of the strain Delcg0424 (Fig. 2d). Thus, Cg0424 and Cg0420 probably have the activities of mannosyltransferase and arabinosyltransferase to catalyze the formation of mannan and arabinomannan, respectively. Moreover, the synthesis of *C. gultamicum* cell-surface polysaccharides probably involves more glycosyltransferases, as the double deletion of *cg0420* and *cg0424* does not abolish the synthesis of mannan or arabinomannan. Deletion of these putative glycosyltransferase genes positively influenced HA synthesis, as HA yield increased by 14.9% with double deletion of *cg0424* and *cg0420* to 6.4 g L^−1^ in shake-flask cultures (Table 1). The optimal engineered *C. glutamicum* strain CgspH-7 underwent fed-batch cultivation. After consuming 166 g L^−1^ glucose, the HA titer reached 34.2 g L^−1^ (Fig. 2e) and the broth turned viscous (Supplementary Table 1).

**Table 1:** Increase HA productivity by gene deletion of *cg0424* and *cg0420*.

Strain	Deleted gene	HA yield (g L^−1^)^a^	Increase in HA yield^a^	*p-*value^b^
CgspH-6	*cg0424*	5.8 ± 0.1	4.8% ± 0.8%	*p* = 0.009
CgspH-7	*cg0424* and *cg0420*	6.4 ± 0.1	14.9% ± 1.0%	*p* = 0.001

^a^The increase in HA yields of strain of CgspH-6 and CgspH-7 were compared to the HA yield of strain CgspH-2 presented in Fig. 1e. All the data are expressed as the mean ± SD from three (*n* = 3) biologically independent replicates.

^b^Statistical evaluation (*p-*value) compared to strain CgspH-2 was performed by two-sided *t*-test.

### HA accumulation encapsulates *C. glutamicum* cells

**Fig. 3:**
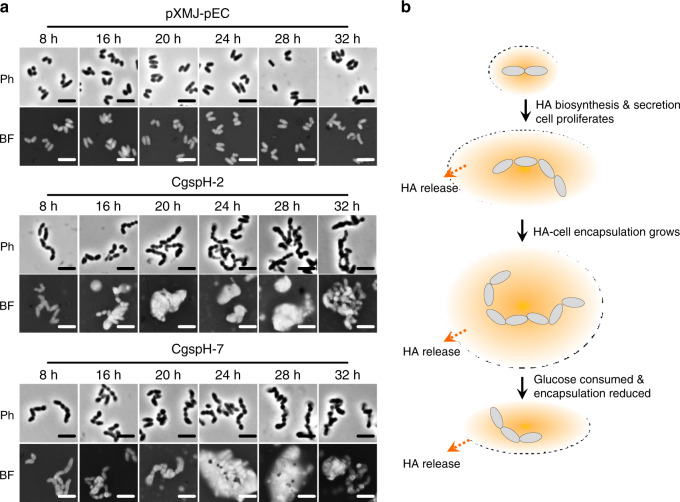
Encapsulation of *C. glutamicum* cells by secreted HA. **a** Representative micrographs of *C. glutamicum* cells producing HA. Strains were cultivated in shake-flasks. Cells were collected at designated time points for visualization by phase-contrast microscopy or under a bright field after negative staining with 100 mg mL^−1^ water-soluble nigrosin for 5 minutes. Ph, phase-contrast microscopy; BF, bright-field microscopy. Scale bar, 2.5 μm. Microcopy experiments were performed three times independently with similar results. **b** A proposed model of HA secretion, encapsulation and release. Source data underlying Fig. 3a are provided as a Source data file.

HA is produced as the main component of some bacterial capsules and is attached to the cell surface to provide protection and virulence. To determine how HA is released into culture broth and its distribution on the *C. glutamicum* surface, we visualized the HA-producing strains CgspH-2 and CgspH-7 by phase-contrast microscopy and found that both strains exhibited cell aggregation along with HA accumulation (Fig. 3a). Moreover, the engineered *C. glutamicum* strains showed a chain-like morphology (Fig. 3a and Supplementary Fig. 6) resembling that of the native HA-producing *Streptococcus* species. To visualize HA distribution, we performed negative staining with nigrosin, a classical capsule-staining dye^42^. Secreted HA formed a capsule-like layer, with HA-producing cells encapsulated (Fig. 3a), whereas no similar structure was observed in the control strain *C. glutamicum* pXMJ-pEC carrying blank vectors. As a consequence, we proposed a model of the encapsulation process (Fig. 3b). HA molecules were not secreted directly into the medium but rather knitted into a slime layer encapsulating the dividing cells. Additionally, the HA molecules are gradually released from the outer layer and dissolved in the culture broth. In the presence of adequate glucose, the size of the cell capsule grow continuously (Fig. 3a between time points 16 and 28 hour), whereas decreased glucose availability at the late growth phase made HA secretion slow down and HA encapsulation decay (Fig. 3a, time point 32 hour).

### The HA capsule layer restricts nutrient uptake

**Fig. 4:**
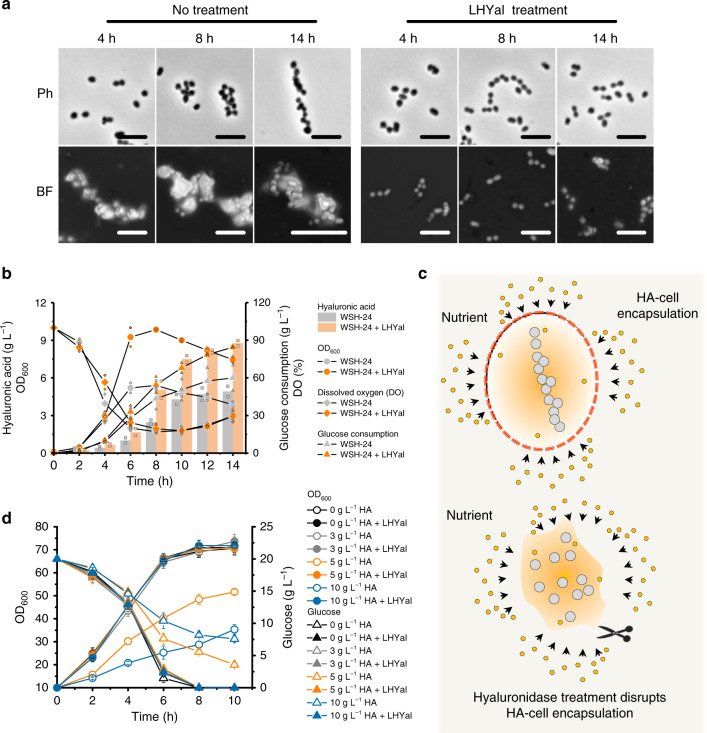
The HA encapsulation inhibits cell growth and glucose uptake. **a** Representative micrographs of HA-producing *S. equi* subsp. *zooepidemicus* WSH-24 cultivated in a 3-L fermenter (refer to **b** for the cultivation details) in the presence or absence of 6000 U mL^−1^ of LHYal. Ph, phase-contrast microscopy; BF, bright-field microscopy. Scale bar, 2.5 μm. Microcopy experiments were performed two times independently with similar results. **b** *S. equi* subsp. *zooepidemicus* WSH-24 cultivated by fed-batch cultivation in a 3-L fermenter to produce HA in the presence of an initial glucose concentration at 70 g L^−1^ and post-inoculation glucose concentration at between 10 and 15 g L^−1^. Total consumed glucose instead of real-time glucose concentration was plotted. LHYal (6000 U mL^−1^, final concentration) was added at the beginning of cultivation. **c** A schematic showing HA encapsulation hinders nutrient uptake. LHYal inhibits HA encapsulation and increases nutrient availability. **d** Effect of HA dissolved in culture broth (weight average MW 1.3 MDa) on glucose uptake by wild-type *C. glutamicum* ATCC 13032. **b** The data are expressed as the mean from two (*n* = 2) biologically independent replicates. Each biological replicate of HA concentration assay is presented as the mean value of three technical repeats. The values of each biologically independent replicate are indicated with same-colored symbols with smaller size. **d** The data are expressed as the mean ± SD from three (*n* = 3) biologically independent replicates. Source data underlying Fig. 4a, b and d are provided as a Source data file.

The HA capsule layer of *Streptococcus* plays important roles in resisting phagocytosis and protecting the cells from desiccation and drying^3^. However, the influence of the HA capsule layer on cell metabolism remains unclear. Therefore, we used recombinant LHYal with high substrate specificity to disrupt the HA capsule layer of the native HA producer *S. equi* subsp. *zooepidemicus* WSH-24 to study the impact of HA capsule on cell metabolism^9^. Supplementation with LHYal (6000 U mL^−1^) disrupted the HA capsule of *S. equi* subsp. *zooepidemicus* (Fig. 4a). Additionally, the disruption of capsule sped up glucose consumption, stimulated cell growth and significantly increased HA yield (Fig. 4b). This result demonstrates that the presence of an HA capsule inhibited nutrient uptake and cell growth. The influence could be relieved by disruption of cell encapsulation by LHYal treatment (Fig. 4c). Moreover, we supplied different concentrations of high-MW HA (1.3 MDa) to cultures of *C. glutamicum* ATCC 13032, a non-HA producer, to examine the impact of HA that released into culture broth on cell growth and metabolism. In order to represent better the natural phenomenon, the HA supplemented here is produced by the natural HA producer *S. equi* subsp. *zooepidemicus*. We found significant decreases in cell growth and glucose consumption in the presence of 5 g L^−1^ HA and 10 g L^−1^ HA (Fig. 4d). By contrast, supplementing equal concentrations of HA pretreated with 10000 U mL^−1^ of LHYal for 8 hours did not slow down cell growth or glucose consumption (Fig. 4d). These results suggest that increased concentration of high-MW HA released into cultures also restricts nutrient uptake.

### Eliminating the capsule-like layer promotes HA production

**Fig. 5:**
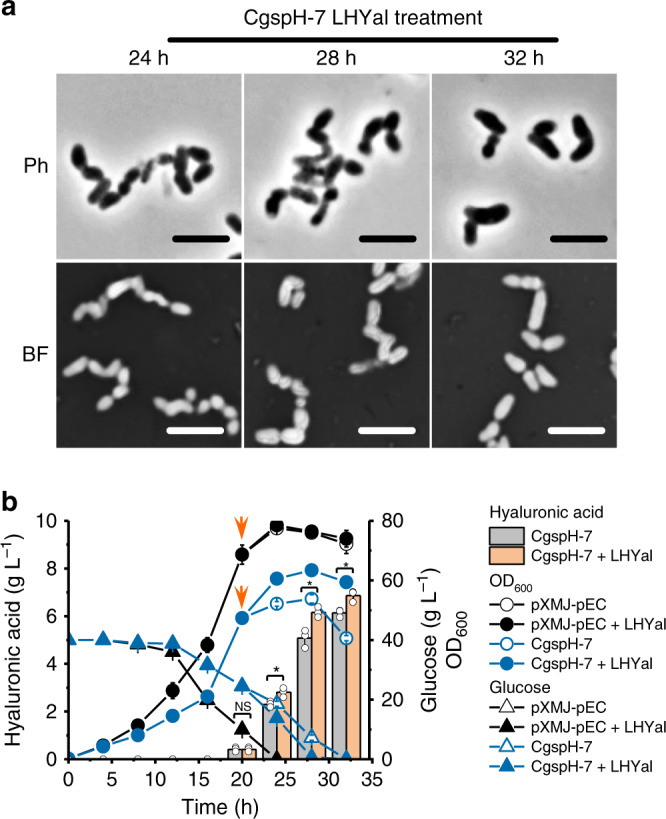
Disruption of HA encapsulation promotes *C. glutamicum* cell growth and HA synthesis. **a** Representative micrographs of CgspH-7 supplemented with 6000 U mL^−1^ LHYal after 20 hours of shake-flask cultivation (refer to **b** for the cultivation details). Cell encapsulation was disrupted by LHYal. Ph, phase-contrast microscopy; BF bright-field microscopy. Scale bar, 2.5 μm. Microcopy experiments were performed three times independently with similar results. **b** LHYal stimulated cell growth, glucose metabolism, and HA production by the CgspH-7 strain. Time point of LHYal supplementation was indicated with arrows. All the data are expressed as the mean ± S.D. from three (*n* = 3) biologically independent replicates. Statistical evaluation (*p-*value) was performed by two-sided *t*-test. *p < 0.05, **p < 0.01, ***p < 0.001; NS, not significant (*p* ≥ 0.05). Source data underlying Fig. 5a, b are provided as a Source data file.

**Fig. 6:**
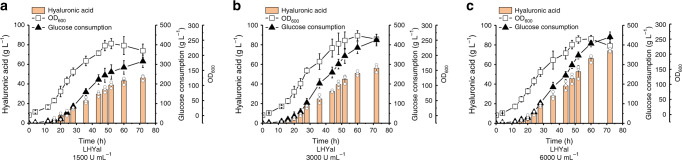
Fed-batch cultivation of CgspH-7 supplemented with different concentrations of LHYal to increase HA bioproduction. 1500 U mL^−1^ (**a**), 3000 U mL^−1^ (**b**), and 6000 U mL^−1^ (**c**) final concentrations of LHYal were supplemented to the fed-batch cultures of CgspH-7. Total consumed glucose, cell density (OD_600_) and HA yields were plotted. During cultivation, glucose was fed manually to maintain a concentration at between 10 and 15 g L^−1^, and ammonia [14% (v/v)] was supplemented to maintain pH at between 6.5 and 7.0. All the data are expressed as the mean ± S.D. from three (*n* = 3) biologically independent replicates. Each biological replicate of HA concentration assay is presented as the mean value of three technical repeats. Source data are provided as a Source data file.

We then determined whether elimination of the capsule-like layer of CgspH-7 would also be beneficial to cell growth and HA production. We supplemented 6000 U mL^−1^ LHYal to CgspH-7 shake-flask cultures upon initiation of HA accumulation. As expected, cell encapsulation did not show up when LHYal was supplemented and the chain-structured cell aggregates partially recovered to the natural rode shape of *C. glutamicum* (Fig. 5a). As a result, the glucose consumption and cell growth of CgspH-7 were enhanced and HA synthesis was accordingly promoted. No such effects were observed on the control strain pXMJ-pEC (Fig. 5b). Furthermore, addition of LHYal to fed-batch cultures of *C. glutamicum* CgspH-7 not only improved HA production, but also resulted in stronger metabolic activities and prolonged exponential phases (Fig. 6a–c). Supplementation of LHYal to a final concentration of 1500, 3000, or 6000 U mL^−1^ increased the total consumed glucose from 166 g L^−1^ (Fig. 2e) to 316 g L^−1^ (Fig. 6a), 424 g L^−1^ (Fig. 6b), and 440 g L^−1^ (Fig. 6c), respectively and increases in the OD_600_ from 172 (Fig. 2e) to 215, 253, and 231 (Fig. 6a–c), respectively. Additionally, this improved HA production to 46.2 g L^−1^ (Fig. 6a), 57.5 g L^−1^ (Fig. 6b), and 74.1 g L^−1^ (Fig. 6c), whereas the weight average MW of HA decreased to 155 kDa, 91 kDa and 53 kDa, respectively (Table 2). These results demonstrated that elimination of the capsule-like layer recovered cell metabolism rate and improved HA production.

**Table 2:** Weight average MW of HA produced in fed-batch cultivations supplemented with different titers of LHYal.

Strain	LHYal (U mL^−1^)	Sampling time poit (hour)	MW (kDa)^a^
CgspH-7	None	48	331 (306, 357)
	1500	72	155 (135, 175)
	3000	72	91 (92, 89)
	6000	72	53 (56, 51)

^a^The data are expressed as the mean from two (*n* = 2) biologically independent replicates which are indicated in brackets.

## Discussion

Construction of biosynthesis pathways of HA and other glycosaminoglycans in genetically engineered microbes represents a green and safe method for production of these invaluable compounds. To construct an efficient HA-producing cell factory, selection of a proper HA synthase is critical, given that HA productivity and MW are largely determined by synthase activity^19^. Most HA synthases are found in vertebrates, whereas bacterial HA synthases are mainly found in the group A *Streptococcus*^3^. Streptococcal HasA has four transmembrane helices and two membrane-associated helices^33–35^; therefore, it is conceivable that the membrane microenvironment influences HasA activity. In the present study, we found that spHasA displayed stronger HA-synthesis activities in *C. glutamicum* and resulted in higher HA accumulation relative to HasA from the other species (Fig. 1b). The differences in the sequences of these enzymes were primarily found in the transmembrane region (Supplementary Fig. 2a), suggesting that spHasA might be better suited to the *C. glutamicum* cytoplasmic membrane. Recently, Westbrook et al.^27^ successfully enhanced the activity of *Streptococcus equisimilis* HasA (sseHasA) in *B. subtilis* by regulating membrane cardiolipin content and distribution. Additionally, previous studies engineered *E. coli* species as alternative HA producers^10,11,43^. However, *E. coli* exhibit a low degree of activity by heterologously expressed HA synthases, possibly because of the inherent incompatibility between HA synthase and the Gram-negative *E. coli* cell structure: *E. coli* cells have two membranes, whereas the type I HA synthase from Gram-positive *Streptococcus* secretes HA chains only through the inner membrane. Protein engineering of HasA^44^, especially changes in the transmembrane region, might represent a possible direction for further improvements in HA production (Supplementary Fig. 2b, c).

In addition to overexpressing UgdA and GlmS for precursor generation (Fig. 1a), deletion of the competing pathway to increase the supply of UDP-glucuronate and UDP-GlcNAc is a commonly used strategy^22,29^. In *C. glutamicum*, there is less availability of UDP-GlcA relative to UDP-GlcNAc, given that overexpression of UgdA improved HA yield by 2-fold (Fig. 1c). Additionally, UDP-GlcNAc is naturally produced for peptidoglycan biosynthesis (Fig. 1a), which is critical for cell survival. UDP-glucose, the precursor of UDP-GlcA, is used to construct the outmost layer of the *C. glutamicum* cell wall^38^, which should be less important to the cell than peptidoglycans for maintaining the cell-envelope structure. A previous report showed that *cg0420* expression enhanced the biosynthesis of extracellular polysaccharides^40^. In the present study, we selected four putative genes encoding glycosyltransferases (*cg0420*, *cg0424*, *cg0419*, and *cg0438*) and located in close proximity to *galU* and *ugdA* for deletion, finding that loss of *cg0424* and *cg0420* reduced cell-surface polysaccharides such as mannan and arabinomannan (Fig. 2d) and enhanced HA production (Table 1) without impairing cell growth (Fig. 2c). These results demonstrated that the enzymes encoded by *cg0420*^40^ and *cg0424* are involved in extracellular polysaccharide synthesis and that the outmost layer of the *C. glutamicum* cell wall^38^ comprising extracellular polysaccharides is likely not essential to cell growth.

The feedback effects of HA accumulation on cell morphology and metabolism have previously been ignored. Here, we found that during the later period of fermentation, cells of the engineered *C. glutamicum* strain were encapsulated following HA accumulation. The formation of a capsule-like layer (Fig. 3a) inhibited cell metabolism and growth (Fig. 4b). The morphology of the *C. glutamicum* CgspH-7 cells resembled the chain shape of native HA-producing *Streptococcus* species (Fig. 4a). Moreover, our results suggest that the capsule of pathogens not only protects cells against various stressors or phages or acts as a virulence factor, but also could adversely affect cell metabolism (Fig. 4b). This could explain why capsule formation occurs under so complex regulatory processes^45^.

In conclusion, optimization of the HA-biosynthesis pathway, inactivation of extracellular polysaccharide biosynthesis, destruction of cell encapsulation and resolving the mass-transfer bottleneck by supplementation with LHYal resulted in an efficient *C. glutamicum* cell factory and a fermentation approach. This engineered strain allowed generation of an average of 74.1 g L^−1^ HA with an MW of 53 kDa in 5-L fed-batch cultures (Fig. 6c and Table 2). The results and strategies reported here are likely applicable to the production of other biopolymers.

## Methods

### Plasmids and strains

Plasmids and strains used in this study are listed in Supplementary Data 1 and Supplementary Data 2. We used *E. coli* JM109 for plasmid amplification and recombinant plasmid construction. *C. glutamicum* ATCC 13032 was used as the parental strain for breeding all engineered *C. glutamicum* strains. HA synthase genes (*hasA*) were amplified from the genome of *S. equi* subsp. *zooepidemicus* (se) or synthesized by GENEWIZ (Suzhou, China) according to the published genome sequences of *Streptococcus pyogenes* (sp), *Streptococcus uberis* (su) and *Pasteurella multocida* (pm). The *hasA* genes were prefixed with “se”, “sp”, “su”, and “pm” in order to indicate species origins.

After amplification with the designated primers sehasA-F and sehasA-R, sphasA-F and sphasA-R, suhasA-F and suhasA-R and pmhasA-F and pmhasA-R (Supplementary Data 3), *hasA* genes were ligated into HindIII/BamHI linearized pXMJ19 using the T5 exonuclease DNA assembly (TDEA) method^46^ to generate the plasmids pXMJ19-sehasA, pXMJ19-sphasA, pXMJ19-suhasA, and pXMJ19-pmhasA. With the  former three  plasmids as templates, the linear form of plasmids pXMJ19-sphasA-6His, pXMJ19-sehasA-6His, pXMJ19-suhasA-6His, pXMJ19-sehasA^Thr7-Met25^ were generated via PCR using designated primers listed in Supplementary Data 3. The PCR products carrying homogenous 5′ and 3′ terminals (included in the designed primers) were transformed into *E. coli* JM109 after purification and cyclized by the endogenous DNA recombinases. The plasmid pXMJ19-sehasA^Thr7-Met25^-6His was constructed by PCR in the same way using pXMJ19-sehasA^Thr7-Met25^ as template. Genes encoding enzymes capable of synthesizing HA building blocks, including *galU* (encoding glucose-1-phosphate uridylyltransferase), *ugdA* (encoding UDP-glucose 6-dehydrogenase), *glmM* (encoding phosphoglucosamine mutase), *glmS* (encoding l-glutamine:d-fructose-6-phosphate aminotransferase), *glmU* (encoding bifunctional N-acetylglucosamine-1-phosphate uridyltransferase and glucosamine-1-phosphate acetyltransferase), were amplified from different species, including *E. coli* MG1655 (ec), *P. putida* KT2440 (pt), *S. equi* subsp. *zooepidemicus* (se) and *C. glutamicum* ATCC13032 (cg), using the designated primers listed in Supplementary Data 3. Additionally, we cloned the *ugdA* genes located in the heparosan synthesis gene cluster of *E. coli* O10:K5:H4 and *E. coli* Nissle 1917. The primers and the amplified genes were prefixed with “ec”, “pt”, “se”, “cg”, “eco”, and “ecn” in order to indicate their species origins. These genes were assembled individually or in specified combinations into the pEC-XK99E plasmid using the TEDA method^46^.

Homologous regions with a length of ~750 bp were amplified upstream and downstream of *cg0420* and *cg0424* using primers 0420-up-F/0420-up-R, 0420-down-F/0420-down-R, and 0424-up-F/0424-up-R, 0424-down-F/0424-down-R, respectively. The homologous regions were combined with the EcoRI/BamHI linearized pK18mobSacB plasmid with to create pK18mobSacB-0420 and pK18mobSacB-0424, which were subsequently used for gene deletions of *cg0420* and *cg0424*.

All plasmids were transferred to *C. glutamicum* ATCC 13032 by electroporation (200 Ω, 12.5 kV cm^−1^, pulse duration 4 ms) and a subsequent heat shock (46 °C for 6 minutes in BHIS medium)^47^. Recombinant strains were selected with kanamycin resistance. In the same way, integrations of the plasmids pK18mobSacB-0420 and pK18mobSacB-0424 into the chromosome were selected with kanamycin resistance. Segregation of the plasmids from chromosome was counter-selected with sucrose-induced lethality^47^. Loss of the gene *cg0420* or *cg0424* was confirmed with colony PCR and DNA sequencing.

### Medium and cultivation

*E. coli* was cultivated in Luria-Bertani medium (tryptone 10 g L^−1^, NaCl 10 g L^−1^, yeast extract 5 g L^−1^, pH 7.0) at 37 °C, and *C. glutamicum* was cultivated at 30 °C in BHIS medium [37 g L^−1^ Brain Heart Infusion (Difco), 91 g L^−1^ sorbitol]. Competent *C. glutamicum* cells for electroporation were prepared by cultivating *C. glutamicum* strains in BHIS medium to OD_600_ of 1.5 and washing cells three times with 10% (v/v) glycerol^47^. Chloramphenicol (15 mg L^−1^), kanamycin (25 mg L^−1^) or sucrose [15% (m/v)] was supplemented, as necessary.

To produce HA in shake-flasks, engineered *C. glutamicum* strains were cultivated at 28 °C with shaking at 200 rpm in a modified glucose-corn steep powder medium^29^. The medium contained 40 g L^−1^ glucose, 20 g L^−1^ corn steep  powder, 20 g L^−1^ (NH4)_2_SO_4_, 1 g L^−1^ KH_2_PO_4_, 1 g L^−1^ K_2_HPO_4_, 0.25 g L^−1^ MgSO_4_, and 42 g L^−1^ MOPS [3-(N-morpholino) propanesulfonic acid]. The initial  optical density at 600 nm (OD_600_) of the shake-flask cultivation was 0.2, and isopropyl‐β‐d‐thiogalactoside (IPTG) was added to induce target gene expression at time point 2.5 hour. LHYal was supplemented at time point 20 hour to a final concentration of 6000 U mL^−1^, as necessary.

Fed-batch cultivations of *C. glutamicum* were performed in a 5-L fermenter with 2.5 L of the glucose-corn steep powder medium. Seed cultures were prepared in glucose-corn steep powder medium containing 40 g L^−1^ glucose at 30 °C and shaken at 220 rpm for 8–10 hours. During fed-batch cultivations, glucose [70% (m/v)] was fed according to real-time glucose consumption in order to dynamically maintain its concentration between 10 and 15 g L^−1^. Ammonia solution [14% (v/v)] was automatically fed in order to maintain the culture pH at between 6.5 and 7.0. Air flow was maintained at ~5 vvm. LHYal was supplemented to final concentrations of 1500, 3000, or 6000 U mL^−1^ at time point 20 hour.

*S. equi* subsp. *zooepidemicus *WSH-24 seed culture was grown at 37 °C and shaking at 220 rpm for 14–16 hours in medium containing 20 g L^−1^ glucose, 20 g L^−1^ yeast extract, 2 g L^−1^ MgSO_4_·7H_2_O, 0.1 g L^−1^ MnSO_4_·4H_2_O, 1.5 g L^−1^ Na_2_HPO_4_, 0.64 g L^−1^ NaH_2_PO_4_, 2.0 g L^−1^ KH_2_PO_4_, 0.5 g L^−1^ NaHCO_3_, 20 g L^−1^ CaCO_3_, 200 μg L^−1^ CaCl_2_, 46 μg L^−1^ ZnCl_2_, 19 μg L^−1^ CuSO_4_·5H_2_O, pH 7.2. Seed cultures were inoculated into a 3-L fermenter containing 1.5 L of fermentation medium with a composition of 70 g L^−1^ glucose, 20 g L^−1^ yeast extract, 6.2 g L^−1^ Na_2_HPO_4_, 1.3 g L^−1^ K_2_SO_4_, 2 g L^−1^ MgSO_4_·7H_2_O, 200 μg L^−1^ CaCl_2_, 46 μg L^−1^ ZnCl_2_, 19 μg L^−1^ CuSO_4_·5H_2_O, pH 7.2. For fed-batch cultivation at 37 °C, NaOH (5 M) was automatically fed to maintain pH between 6.9 and 7.1, and air flow was maintained at ~3 vvm. LHYal (final concentration 6000 U mL^−1^) was supplemented at the time of seed inoculation.

### Measurement of cell growth and metabolite concentration

Cell growth was monitored by changes in the OD_600_. Real-time glucose concentration was measured using an M-100 biosensor analyzer (Shenzhen Siemantec Technology, Shenzhen, China). To measure total HA, culture broths were autoclaved at 121 °C for 30 minutes. Cell debris was removed by centrifugation at 16,099 × *g* for 15 minutes. Supernatants were pooled and mixed with four volumes of ice-cold ethanol and HA was precipitated at −30 °C overnight. Insoluble fractions were subsequently collected by centrifugation at 16,099 × *g* for 10 minutes. Residual ethanol was evaporated at room temperature and the semi-dry insoluble fraction was dissolved in deionized water, whereas the water-insoluble fraction was removed by centrifugation. To remove as much impurity, these steps were repeated three times more. After appropriate dilution (5–1000 fold, depending on HA concentration), HA content was determined via carbazole assay using glucuronic acid as an external standard^48^. Supernatants from *C. glutamicum* culture harboring the blank vector pXMJ19 or pXMJ19/pEC-XK99E were used as the negative control. To measure secreted HA, cells were removed from the broth by centrifugation at 16,099 × *g* for 15 minutes and HA was precipitated from the supernatant for measurement, as described above.

### Analysis of *C. glutamicum* extracellular polysaccharides

*C. glutamicum* ATCC 13032 and strains lacking *cg0424* or *cg0420* and *cg0424* were cultivated in CGXII minimal medium^47^ at 28 °C for 48 h. Cells were shaken with glass beads at 200 rpm and room temperature for 1 h to release surface exposed materials including extracellular polysaccharides^38^. Cells were removed by centrifugation at 7155 × *g* for 20 minutes. Supernatants were pooled and hydrolyzed with trifluoroacetic acid at 110 °C for 2 hours. Hydrolysates containing reducing sugars were dried in vacuum chamber at 65 °C for 2 hours. Reducing sugars generated from hydrolysis of extracellular polysaccharides reacted with 1-phenyl-3-methyl-5-pyrazolone (PMP) at 70 °C for 60 minutes in the NaOH-methanol solution to form the sugar-PMP^49^. The sugar constituent of *C. glutamicum* extracellular polysaccharides was analyzed using Agilent 1200 HPLC system equipped with a SHISEIDO CAPCELL PAK C_18_ column (inner diameter 4.6 mm, length 250 mm, particle size 5 μm). Chemicals were separated in the column using a mobile phase of 0.1 M KH_2_PO_4_ (pH 6.8) [82% (v/v)] and acetonitrile [18% (v/v)] with a flow rate of 1 mL min^−1^. Fractions were detected with absorbance at wavelength of 245 nm.

### Preparation of recombinant leech hyaluronidase

LHYal samples were prepared and purified from recombinant *Pichia pastoris* through Ni-sepharose affinity resin (HisTrap FF column, GE Healthcare)^32^. Purified LHYal was stored at −30 °C until use. Determination of the LHYal glycoside hydrolase activity was performed routinely by the 3,5-dinitrosalicylic acid colorimetric quantification of enzymatically released reducing sugars^32^.

### Western Blot of type I hyaluronan synthase

*C. glutamicum* ATCC13032 expressing C terminally 6× histidine tagged spHasA, seHasA, suHasA, seHasA^Thr7-Met25^ were digested with 20 mg mL^−1^ lysozyme at room temperature for 2 hours and lysed with sonication in buffer containing 50 mM Tris-HCl, 5 mM ethylenediaminetetraacetic acid and 1 mM phenylmethanesulfonyl fluoride. Cell debris was removed by centrifugation at 16,099 × *g* for 15 minutes. After BCA protein concentration assay, equal amount (20 μg) of total protein samples were applied to SDS-PAGE. Afterwards, Western Blot was performed with 1:5000 diluted  YTHXBio ZA004 His-tag mouse monoclonal antibody and 1:10000  diluted horseradish peroxidase labeled YTHXBio ZM03 goat anti-mouse IgG(H + L)-HRP) (YTHX Biotechnology, Beijing, China).

### Analysis of cell morphology by microscopy

For phase-contrast microscopy, cells were loaded on the thin layer of an agarose pad and visualized using an Eclipse Ni-E microscope (Nikon, Tokyo, Japan) equipped with a module for phase-contrast microscopy. To visualize cell encapsulation, cells were negatively stained with 100 mg mL^−1^ water-soluble nigrosin (Sangon Biotech, Shanghai, China), air dried and visualized with Eclipse Ni-E bright field microscope. Micrographs were processed with ImageJ^50^.

### Determination of HA effects on cell metabolism and growth

*C. glutamicum* ATCC 13032 was grown in glucose-corn steep powder medium for 10 hours, followed by centrifugation at 1789 × *g* for 10 minutes and resuspension with fresh glucose-corn steep powder medium containing 20 g L^−1^ glucose and designated concentrations (0, 3, 5, or 10 g L^−1^) of HA [obtained from Bloomage Biotechnology CO, LTD (Jinan, China) with purity higher than 98%] or HA pretreated with 10,000 U mL^−1^ LHYal at room temperature for 8 hours. The initial OD_600_ was set to 10 and cell growth and glucose consumption were measured every 2 hours.

### Measurement HA weight average MW

Supernatant containing HA was isolated, as described above, using ice-cold ethanol precipitation. The crude HA samples were subjected to further purification with anion exchange chromatography via the ÄKTA avant 25 preparative chromatography system equipped with a HiPrep Q HP 16/10 (GE Healthcare, USA) column. Column was equilibrated with 50 mM Tris-HCl (pH 8.0); fractions were gradient eluted with 0–200 mM NaCl and monitored with absorbance at wavelength of 210 nm. Fractions were collected, freeze-dried, dissolved with appropriate amount of water and filtrated through a 0.22 μm membrane. The weight average MW of HA was measured using high-performance size-exclusion chromatography (HPSEC) with multi-angle laser light scattering (MALLS) analysis. Briefly, 100 μL of HA sample was injected into an HPSEC-MALLS system (equipped with Waters 515 HPLC pump, Shodex OHpak SB-806HQ and Shodex OHpak SB-804HQ column series, DAWN HELEOS II MALLS instrument and Optilab dRI detector), fractions were separated in the column series with the mobile phase of 0.02% (m/v) NaN_3_ at 25 °C at a flow rate of 1.0 mL min^−1^. The average value of two measurements was used to calculate the final weight average MW of HA.

### Reporting summary

Further information on research design is available in the Nature Research Reporting Summary linked to this article.

## Supplementary information

Supplementary Information

Reporting Summary

Description of Additional Supplementary Files

Supplementary Data 1

Supplementary Data 2

Supplementary Data 3

Supplementary Data 4

## Data Availability

The authors declare that all data supporting the findings of this study are available within the paper and its supplementary information files. The datasets generated and analyzed during the current study are also available from the corresponding author upon request. The source data underlying Figures 1b–e, 2c–e, 3a, 4a, b, d, 5a, b, 6a–c, and Tables 1 and  2 as well as Supplementary Figures 2b, c, 4a–c, 6 and Supplementary Table 1 are provided as a Source Data file. Source data are provided with this paper.

## Source data

Source Data
