# Supplementary material for: Eliminating the capsule-like layer to promote glucose uptake for hyaluronan production by engineered Corynebacterium glutamicum
Source: Nat Commun. 2020 Jun 19;11:3120. doi: 10.1038/s41467-020-16962-7 (PMC7305114; doi:10.1038/s41467-020-16962-7)
Supplement: Supplementary file 1 — Supplementary Information [file 41467_2020_16962_MOESM1_ESM.pdf]

Supplementary Information

Eliminating the capsule-like layer to promote glucose uptake for hyaluronan production by engineered *Corynebacterium glutamicum*

Wang *et al.*

This file contains

Supplementary Figures

Supplementary Figure 1

Supplementary Figure 2

Supplementary Figure 3

Supplementary Figure 4

Supplementary Figure 5

Supplementary Figure 6

Supplementary Tables

Supplementary Table 1

Supplementary References

Supplementary Figures

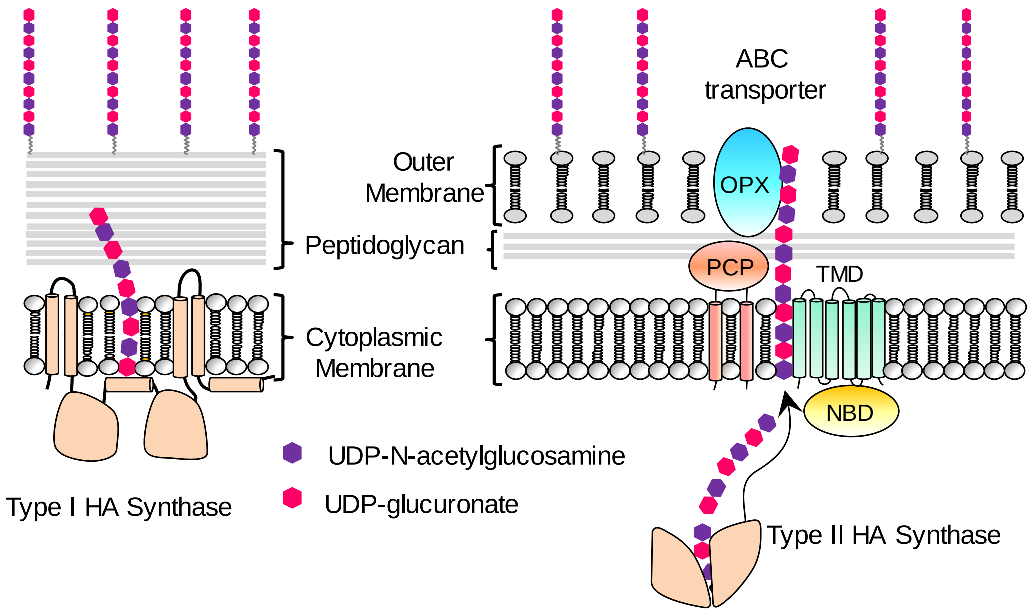

Supplementary Fig. 1 Schematics of two types of hyaluronic acid (HA) synthases. Type I HA synthase is a transmembrane protein which elongates the HA chain as well as secrets it to cell exterior. Type II HA synthase locates in cytoplasm. It has no HA transport capabilities. An independent ABC transporter is required to secret the HA chains[1, 2]. OPX, outer membrane polysaccharide exporter; PCP, polysaccharide co-polymerase; NBD, nucleotide-binding domain; TMD: transmembrane domain.

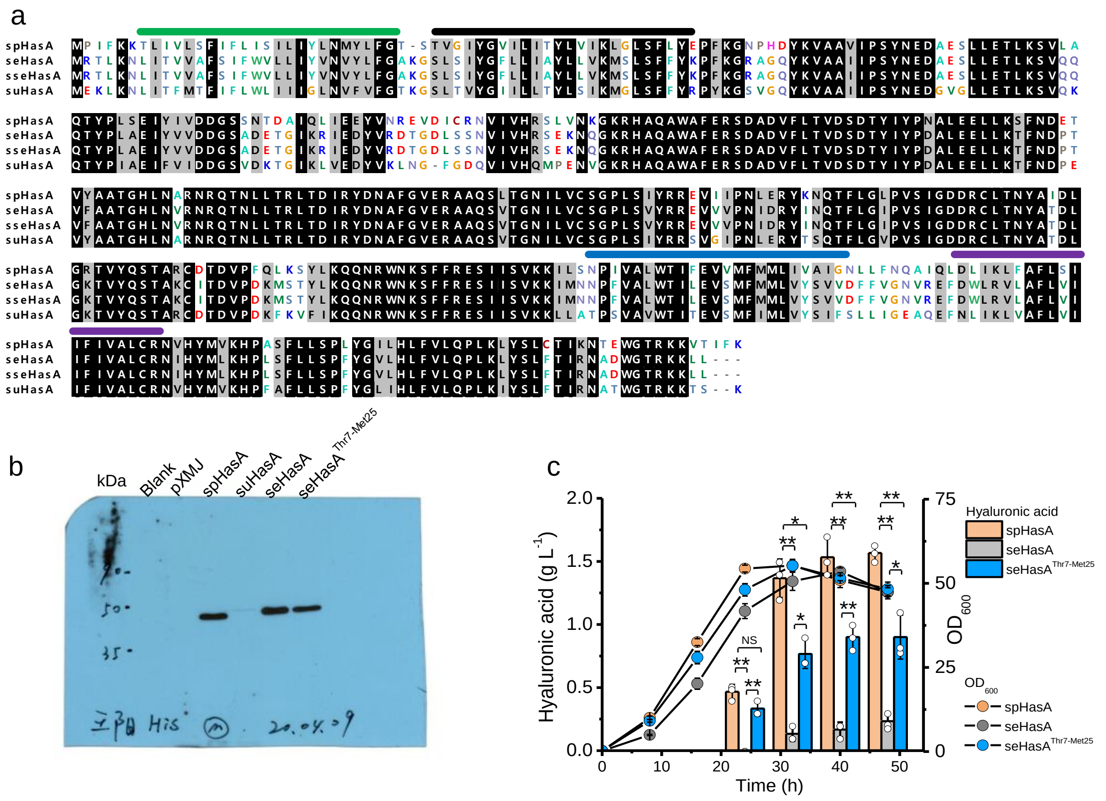

Supplementary Fig. 2 Analysis the protein sequence, expression feature and HA synthesis capabilities of type I HA synthases from streptococcal species. **a** Comparing the protein sequences of *S. pyogenes* (spHasA) and *S. equi subsp. zooepidemicus* HasA (seHasA), *S. uberis* (suHasA) and *Streptococcus equisimilis* HasA (sseHAS). Identical or similar amino acids were shaded by black or gray, respectively. Transmembrane regions were indicated with colored lines. **b** Western Blot analysis of the expression of spHasA, suHasA, seHasA and a seHasA$^{Thr7\text{-}Met25}$ (in this mutant the first transmembrane helix of seHasA was replaced by that of the spHasA). All the HA synthases are C terminally tagged by 6x histidine tag. Western Blot was performed with YTHXBio ZA004 His-tag mouse monoclonal antibody and horseradish peroxidase labeled YTHXBio

ZM03 goat anti-mouse IgG(H+L)-HRP (YTHX Biotechnology ,Beijing, China). **c** Comparing the HA synthesis capabilities of spHasA, seHasA and the seHasA mutant, seHasA$^{Thr7\text{-}Met25}$. In Supplementary Fig. 2c, the data are expressed as the mean ± S.D. from three (n = 3) biologically independent replicates. Statistical evaluation (p -value) was performed by two-sided t-test. *$p < 0.05$, **$p < 0.01$, ***$p < 0.001$; NS, not significant ($p >= 0.05$). Source data underlying Supplementary Fig. 2b and 2c are provided as a Source data file.

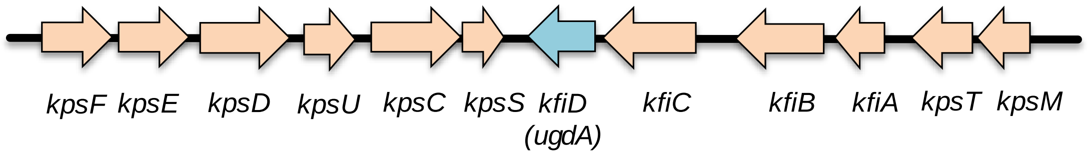

Supplementary Fig. 3 The *ugdA* (*kfiD*) gene locates in the heparosan synthesis gene cluster of *E. coli* O10:K5(L):H4 (eco) or *E. coli* Nissle 1917 (ecn).

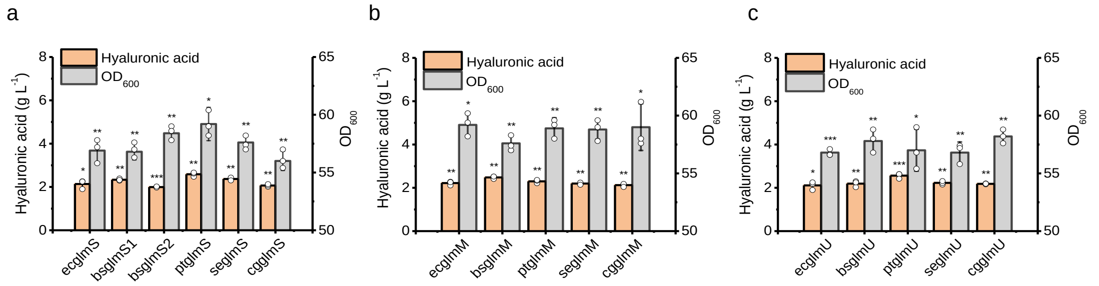

Supplementary Fig. 4 Comparison of the activities of GlmS, GlmM and GlmU from different species on enhancing HA production. **a** GlmS, L-glutamine:D-fructose-6-phosphate aminotransferase. **b** GlmM, phosphoglucosamine mutase. **c** GlmU, glucosamine-1-phosphate N-acetyltransferase. All the data are expressed as the mean ± S.D. from three (n = 3) biologically independent replicates. Statistical evaluation (*p*-value) compared to strain spHasA was performed by two-sided *t*-test. *$p < 0.05$, **$p < 0.01$, ***$p < 0.001$; NS, not significant ($p >= 0.05$). Source data are provided as a Source data file.

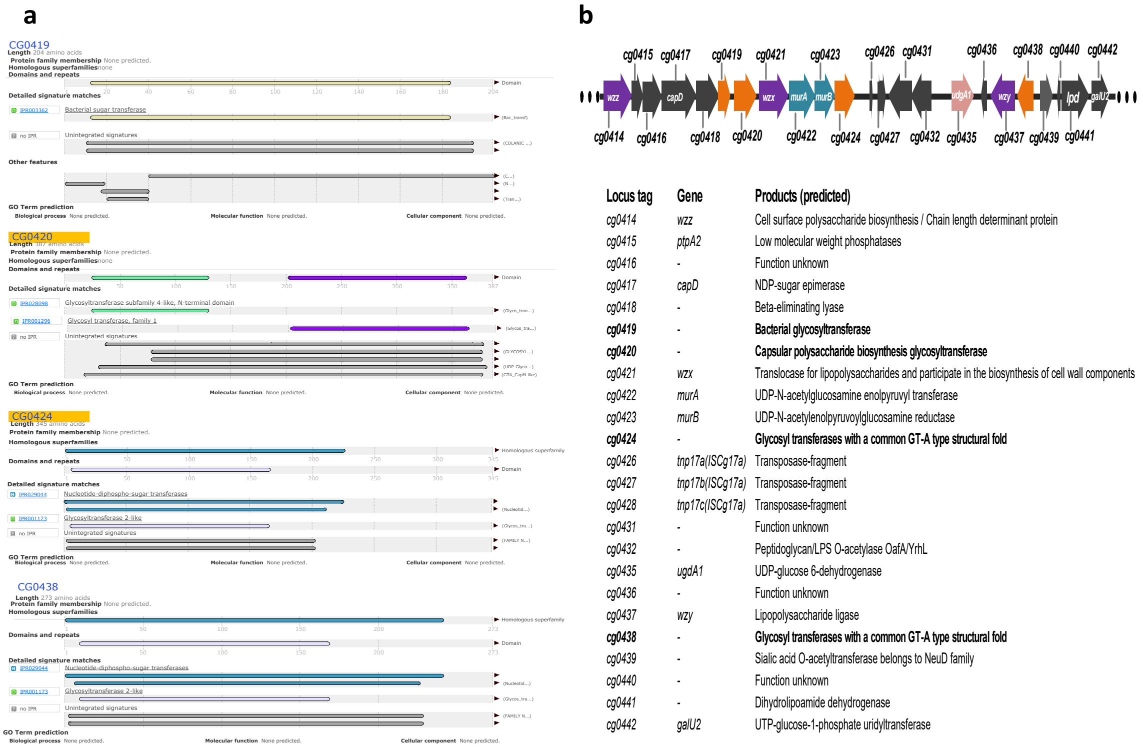

Locus tag	Gene	Products (predicted)
cg0414	*wzz*	Cell surface polysaccharide biosynthesis / Chain length determinant protein
cg0415	*ptpA2*	Low molecular weight phosphatases
cg0416	-	Function unknown
cg0417	*capD*	NDP-sugar epimerase
cg0418	-	Beta-eliminating lyase
cg0419	-	**Bacterial glycosyltransferase**
cg0420	-	**Capsular polysaccharide biosynthesis glycosyltransferase**
cg0421	*wzx*	Translocase for lipopolysaccharides and participate in the biosynthesis of cell wall components
cg0422	*murA*	UDP-N-acetylglucosamine enolpyruvyl transferase
cg0423	*murB*	UDP-N-acetylenolpyruvoylglucosamine reductase
cg0424	-	**Glycosyl transferases with a common GT-A type structural fold**
cg0426	*tnp17a(ISCg17a)*	Transposase-fragment
cg0427	*tnp17b(ISCg17a)*	Transposase-fragment
cg0428	*tnp17c(ISCg17a)*	Transposase-fragment
cg0431	-	Function unknown
cg0432	-	Peptidoglycan/LPS O-acetylase OafA/YrhL
cg0435	*ugdA1*	UDP-glucose 6-dehydrogenase
cg0436	-	Function unknown
cg0437	*wzy*	Lipopolysaccharide ligase
cg0438	-	**Glycosyl transferases with a common GT-A type structural fold**
cg0439	-	Sialic acid O-acetyltransferase belongs to NeuD family
cg0440	-	Function unknown
cg0441	-	Dihydrolipoamide dehydrogenase
cg0442	*galU2*	UTP-glucose-1-phosphate uridyltransferase

Supplementary Fig. 5 Putative extracellular polysaccharides synthases of *C. glutamicum*. **a** The InterPro[3] predicted glycotransferase functions of Cg0419, Cg0420, Cg0424 and Cg0438. **b** The genomic context of *cg0419*, *cg0420*, *cg0424*, *cg0438* and the predicted functions of the neighboring genes.

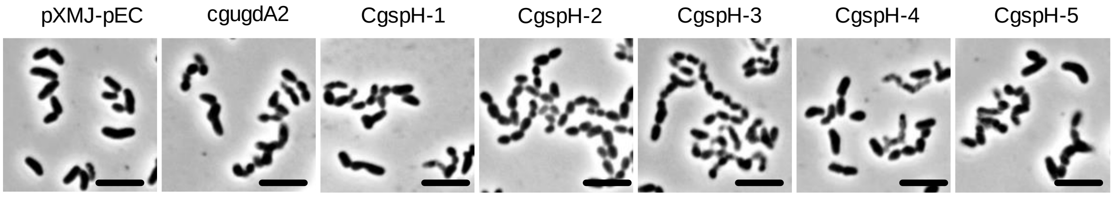

Supplementary Fig. 6. Representative micrographs of the morphology of the *C. glutamicum* strain with different HA synthesis capabilities. Strains were cultivated in shake flasks for 28 hours and examined under phase contrast microscope. Scale bar, 2.5 μm. Microcopy experiments were performed three times independently with similar results. Source data are provided as a Source data file.

Supplementary Tables

Supplementary Table 1 Viscosity of culture broths supplemented with different titers of LHYal

Strain	LHYal (U mL^{-1})	Sampling time (h)	Viscosity [a] (cP)
CgspH-7	None	48	335 (320, 349)
	1,500	72	239 (254, 223)
	3,000	72	79 (91, 66)
	6,000	72	5 (5, 4)

[a] The viscosity of culture broths was measured at 16 °C with Shanghai FangRui NDJ-5S viscometer (Shanghai, China). The data are expressed as the mean from two (n = 2) biologically independent replicates. Each biological replicate of viscosity in brackets was presented as the mean value of three technical repeats.

Supplementary References

1. Willis, L.M. & Whitfield, C. Structure, biosynthesis, and function of bacterial capsular polysaccharides synthesized by ABC transporter-dependent pathways. *Carbohydr Res* **378**, 35-44 (2013).
2. Willis, L.M. & Whitfield, C. KpsC and KpsS are retaining 3-deoxy-D-manno-oct-2-ulosonic acid (Kdo) transferases involved in synthesis of bacterial capsules. *Proc Natl Acad Sci U S A* **110**, 20753-20758 (2013).
3. Mitchell A.L. *et al*. InterPro in 2019: improving coverage, classification

and access to protein sequence annotations. *Nucleic Acids Res* **47**, https://doi.org/ 10.1093/nar/gky1100 (2019).
