# Supplementary material for: Eliminating the capsule-like layer to promote glucose uptake for hyaluronan production by engineered Corynebacterium glutamicum
Source: Nat Commun. 2020 Jun 19;11:3120. doi: 10.1038/s41467-020-16962-7 (PMC7305114; doi:10.1038/s41467-020-16962-7)
Supplement: Supplementary file 2 — Reporting Summary [file 41467_2020_16962_MOESM2_ESM.pdf]

nature research

Corresponding author(s): Zhen Kang

Last updated by author(s): May 27, 2020

Reporting Summary

Nature Research wishes to improve the reproducibility of the work that we publish. This form provides structure for consistency and transparency in reporting. For further information on Nature Research policies, see our Editorial Policies and the Editorial Policy Checklist.

Statistics

For all statistical analyses, confirm that the following items are present in the figure legend, table legend, main text, or Methods section.

n/a	Confirmed	
☐	☒	The exact sample size (*n*) for each experimental group/condition, given as a discrete number and unit of measurement
☐	☒	A statement on whether measurements were taken from distinct samples or whether the same sample was measured repeatedly
☐	☒	The statistical test(s) used AND whether they are one- or two-sided<br>*Only common tests should be described solely by name; describe more complex techniques in the Methods section.*
☒	☐	A description of all covariates tested
☒	☐	A description of any assumptions or corrections, such as tests of normality and adjustment for multiple comparisons
☐	☒	A full description of the statistical parameters including central tendency (e.g. means) or other basic estimates (e.g. regression coefficient) AND variation (e.g. standard deviation) or associated estimates of uncertainty (e.g. confidence intervals)
☐	☒	For null hypothesis testing, the test statistic (e.g. *F*, *t*, *r*) with confidence intervals, effect sizes, degrees of freedom and *P* value noted<br>*Give P values as exact values whenever suitable.*
☒	☐	For Bayesian analysis, information on the choice of priors and Markov chain Monte Carlo settings
☒	☐	For hierarchical and complex designs, identification of the appropriate level for tests and full reporting of outcomes
☒	☐	Estimates of effect sizes (e.g. Cohen's *d*, Pearson's *r*), indicating how they were calculated

Our web collection on statistics for biologists contains articles on many of the points above.

Software and code

Policy information about availability of computer code

Data collection	NCBI Nucleotide Database, NCBI Protein Database , NCBI Nucleotide Blast and NCBI Protein Blast were used for collecting the gene and protein sequences.
Data analysis	Microsoft Excel 2019, Power Point 2019, Origin 2016 and Image J were used for date analysis

For manuscripts utilizing custom algorithms or software that are central to the research but not yet described in published literature, software must be made available to editors and reviewers. We strongly encourage code deposition in a community repository (e.g. GitHub). See the Nature Research guidelines for submitting code & software for further information.

Data

Policy information about availability of data

All manuscripts must include a data availability statement. This statement should provide the following information, where applicable:

- Accession codes, unique identifiers, or web links for publicly available datasets
- A list of figures that have associated raw data
- A description of any restrictions on data availability

The authors declare that all data supporting the findings of this study are available within the paper and its supplementary information files. A reporting summary for this Article is available as a Supplementary Information file. The datasets generated and analyzed during the current study are also available from the corresponding author upon request. The source data underlying Figures 1b-1e, 2c-2e, 3a, 4a, 4b, 4d, 5a, 5b, 6a-6c, and Table 1 and Table 2 as well as Supplementary Figure 2b, 2c, 4a-4c, 6 and Supplementary Table 1 are provided as a Source Data file.

Field-specific reporting

Please select the one below that is the best fit for your research. If you are not sure, read the appropriate sections before making your selection.

- [x] Life sciences
- [ ] Behavioural & social sciences
- [ ] Ecological, evolutionary & environmental sciences

For a reference copy of the document with all sections, see nature.com/documents/nr-reporting-summary-flat.pdf

Life sciences study design

All studies must disclose on these points even when the disclosure is negative.

Sample size	No sample sized calculation was performed. This experiment was performed on bacteria cultivation in either shake flasks or in fermentors. Sample size was 3 for all shake flasks cultivation, 3 for C. glutamicum fed-batch cultivation and 2 for S. equi subsp. zooepidemicus fed-batch cultivation.
Data exclusions	No data was excluded.
Replication	Each experiment was performed with at least two biologically independent replicates, as indicated in the figure legends, to ensure reliability. We have encountered no problems with reproducibility.
Randomization	Single colonies were picked randomly and propagated as bacterial cultures. Representative micrographs in Fig. 3a, 4a, 5a and Supplementary Fig. 6 were selected randomly from biologically independent replicates and presented in the figures.
Blinding	Blinding was not relevant to this study, because the analysis was carried out entirely on bacteria.

Reporting for specific materials, systems and methods

We require information from authors about some types of materials, experimental systems and methods used in many studies. Here, indicate whether each material, system or method listed is relevant to your study. If you are not sure if a list item applies to your research, read the appropriate section before selecting a response.

Materials & experimental systems

n/a	Involved in the study	
[ ]	[x]	Antibodies
[x]	[ ]	Eukaryotic cell lines
[x]	[ ]	Palaeontology and archaeology
[x]	[ ]	Animals and other organisms
[x]	[ ]	Human research participants
[x]	[ ]	Clinical data
[x]	[ ]	Dual use research of concern

Methods

n/a	Involved in the study	
[x]	[ ]	ChIP-seq
[x]	[ ]	Flow cytometry
[x]	[ ]	MRI-based neuroimaging

Antibodies

Antibodies used	YTHXBio ZA004, His-tag mouse monoclonal antibody; YTHXBio ZM03, goat anti-mouse IgG(H+L)-HRP
Validation	Primary antibody, YTHXBio ZA004, His-tag mouse monoclonal antibody. The following validation information is translated from Chinese description on the web page of the antibody supplier, YTHX Biotechnology (Beijing) CO., Ltd. Immunogen: synthetic HHHHHH coupled with KLH. Product description: His refers to a fusion tag composed of six histidines, which is widely used in the detection and purification of fusion proteins. The advantages of His tags are: His tags have a small molecular weight and generally do not affect the function of the target protein. His tag has strong adsorption to solid nickel, and the His-tagged proteins can be purified with a nickel column even under denatured conditions. Our His-tag mouse monoclonal antibody is highly purified monoclonal antibodies that can recognize His tag on the N-terminal or C-terminal fusion proteins with high specificity. Recommended dilution: Western Blot (WB), 1:1000-1:10000; Immunofluorescence: 1:100-1:1000, Immunoprecipitation: 1: 100-1:1000. Solution composition: 0.01M PBS (PH7.4), 0.1% BSA, 0.02% sodium azide, 50% glycerol. Precautions: 1, Please choose a secondary anti-mouse antibody, such as Goat anti-Mouse IgG(H+L)-HRP; 2, Please determine the best antibody dilution according to the experiment; 3, This product is limited to scientific research and cannot be used for clinical diagnosis. Secondary antibody, YTHXBio ZM03, Goat anti-Mouse IgG(H+L)-HRP. The following validation information is obtained from the web page of the antibody supplier, YTHX Biotechnology (Beijing) CO., Ltd. The secondary antibody Goat anti-Mouse IgG(H+L)-HRP is improved based on the original production and have a strong reaction with the IgG heavy and light chains. Meanwhile, the secondary antibody has low reaction with human IgG through adsorption human serum protein. Compare with ordinary secondary antibody, our antibody with high sensitivity and specificity and so on. And application for Western Blot, enzyme-linked immunosorbent assay (ELISA) and other experiments. Application: The source of resistance for an anti-immune mice, respectively,Western blot, ELISA and other experiments. Recommended dilutions: WB:1:1000-1:10000. ELISA:1:5000-1:20000

(Note: This kit without enzyme substrate reagent.) Size(mg/mL): 100 !l/mL. Storage instructions: Goat anti-Mouse IgG(H+L)-HRP is stable for one year at -20°C. Avoid repeated freezing and thawing cycles.
