# Supplementary material for: Eliminating the capsule-like layer to promote glucose uptake for hyaluronan production by engineered Corynebacterium glutamicum
Source: Nat Commun. 2020 Jun 19;11:3120. doi: 10.1038/s41467-020-16962-7 (PMC7305114; doi:10.1038/s41467-020-16962-7)
Supplement: Supplementary file 3 — Description of Additional Supplementary Files [file 41467_2020_16962_MOESM3_ESM.pdf]

Description of Additional Supplementary Files

File Name: Supplementary Data 1

Description: Plasmids used in this study.

File Name: Supplementary Data 2

Description: Strains used in this study.

File Name: Supplementary Data 3

Description: Primers used in this study.

File Name: Supplementary Data 4

Description: Gene sequences and protein sequences of the following enzymes

HasA: HA synthase genes;
GalU: Glucose-1-phosphate uridylyltransferase;
UgdA: UDP-glucose 6-dehydrogenase;
GlmS: L-Glutamine-D-fructose-6-phosphate aminotransferase;
GlmM: Phosphoglucosamine mutase;
GlmU: UDP-N-acetylglucosamine pyrophosphorylase / Glucosamine-1-phosphate N-acetyltransferase;
Cg0420: Putative glycosyltransferase of *C. glutamicum* ATCC 13032;
Cg0424: Putative glycosyltransferase of *C. glutamicum* ATCC 13032.
